# Supplementary material for: Pro-Inflammatory Serum Amyloid a Stimulates Renal Dysfunction and Enhances Atherosclerosis in Apo E-Deficient Mice
Source: Int J Mol Sci. 2021 Nov 22;22(22):12582. doi: 10.3390/ijms222212582 (PMC8623330; doi:10.3390/ijms222212582)

Pro-inflammatory Serum Amyloid A-stimulates Renal and Vascular Dysfunction in Apo E-Deficient Mice

Antony Gao, Sameesh Gupta, Han Shi, Yuyang Liu, Angie L. Schroder, Paul K. Witting and Gulfam Ahmad*

Redox Biology Group, Discipline of Pathology, Faculty of Medicine and Health, Charles Perkins Centre, The University of Sydney

Supplementary data

*Address all correspondence to: Dr Gulfam Ahmad (PhD) – current address: School of Medical Sciences, Faculty of Medicine and Health, Charles Perkins Centre, The University of Sydney, NSW, 2006 Australia. E-mail: gulfam.ahmad@sydney.edu.au

Supplementary figures legends

Figure S1. Medullary Nrf-2 in response to SAA in mice renal tissue. (A) Male ApoE -/- mice were randomly allocated into 2 experimental groups: vehicle control (administered 100 µL PBS) and SAA (receiving 120 µg SAA protein/mL). Four weeks after cessation of treatment, kidney tissues were fixed *in situ* then harvested, embedded and sectioned (5 µm) . Renal sections were dewaxed and rehydrated before undergoing heat-induced antigen retrieval. Medullary Nrf2 expression was assessed using immunofluorescence microscopy. Slides were visualised at 40x magnification (scale bar = 20 µm); at least 4 fields of view were obtained for each sample. Nuclei were stained with DAPI (blue) and Nrf2 with an appropriate Opal fluorophore (red). White arrows show regions of relatively high tubular Nrf2+ immunostaining. Insets show higher magnification images (scale bar = 10 µm), Nrf2+ staining was mixed with Nrf2 co-localised to nuclei with residual cytoplasmic staining. Representative images show cortical fields from n=5 (Control), n=6 (SAA). **(B)** Immunostaining was quantified using a mean staining intensity for each field of view and averaged for each sample. Data shown represent relative mean ± SD. *Relative to control group; $p < 0.05$.

Figure S2. Medullary NF-kB p-p65 following SAA administration. (A) Male ApoE -/- mice were randomly allocated to vehicle control (administered 100 µL PBS) and SAA groups (receiving 120 µg SAA protein/mL). Kidney tissue was harvested 4 weeks after cessation of treatment and fixed in situ before embedding and sectioning (5 µm). Renal sections were dewaxed, then rehydrated before undergoing heat-induced

antigen retrieval. NF-kB p-p65 expression was assessed using immunofluorescence microscopy. Slides were visualised at 40x magnification (scale bar = 20 µm). At least 4 fields of view were obtained for each sample. Nuclei were stained with DAPI (blue) and NF-kB p-p65 with an appropriate Opal fluorophore (red). White arrows indicate NF-kB+ staining localised to renal epithelial cells. Insets show higher magnification (scale bar = 10 µm) images of renal tubular epithelial cells with NF-kB p-p65+ staining largely colocalised to nuclei with some residual cytoplasmic staining. Representative images show cortical fields from n=5 (control), n=6 (SAA). **(B)** Immunostaining was quantified using a mean staining intensity for each field of view and averaged for each sample. Data shown represent relative mean ± SD. **Relative to control group; $p < 0.001$.

Figure S3. p-p38 MAPK medullary expression following SAA administration. (A) Male ApoE -/- mice were randomly allocated to vehicle control (administered 100 µL PBS) and SAA groups (receiving 120 µg SAA protein/mL). Kidney tissue was harvested 4 weeks after cessation of treatment and fixed in situ before embedding and sectioning (5 µm). Renal sections were dewaxed, then rehydrated before undergoing heat-induced antigen retrieval. NF-kB p-p65 expression was assessed using immunofluorescence microscopy. Slides were visualised at 40x magnification (scale bar = 20 µm). At least 4 fields of view were obtained for each sample. Nuclei were stained with DAPI (blue) and p-p38 MAPK with an appropriate Opal fluorophore (red). White arrows indicate p-p38 MAPK+ staining localised to renal epithelial cells. Insets show higher magnification (scale bar = 10 µm) images of renal tubular epithelial cells with p-p38 MAPK+ staining largely colocalised to nuclei with some residual cytoplasmic staining. Representative images show cortical fields from n=5 (control), n=6 (SAA). **(B)** Immunostaining was quantified using a mean staining intensity for each field of view and averaged for each sample. Data shown represent relative mean ± SD. **Relative to control group; $p < 0.001$.

Figure S4. Medullary localisation of IFN-γ following SAA administration. (A) Male ApoE -/- mice were randomly allocated to vehicle control (administered 100 µL PBS) and SAA groups (receiving 120 µg SAA protein/mL). Kidney tissue was harvested 4 weeks after cessation of treatment and fixed in situ before embedding and sectioning (5 µm). Renal sections were dewaxed, then rehydrated before undergoing heat-induced antigen retrieval. IFN-γ localisation was assessed using immunofluorescence microscopy. Slides were visualised at 40x magnification (scale bar = 20 µm). At least 4 fields of view were obtained for each sample. Nuclei were stained with DAPI (blue) and IFN-γ with an appropriate Opal fluorophore (red). White arrows indicate IFN-γ staining primarily localised to renal epithelial cells. Insets show higher magnification (scale bar = 10 µm) images of renal tubular epithelial cells with IFN-γ staining largely colocalised to nuclei with some residual cytoplasmic staining. Representative images

show cortical fields from n=5 (control), n=6 (SAA). **B)** Immunostaining was quantified using a mean staining intensity for each field of view and averaged for each sample. Data shown represent relative mean ± SD. **Relative to control group; $p < 0.001$.

Figure S1

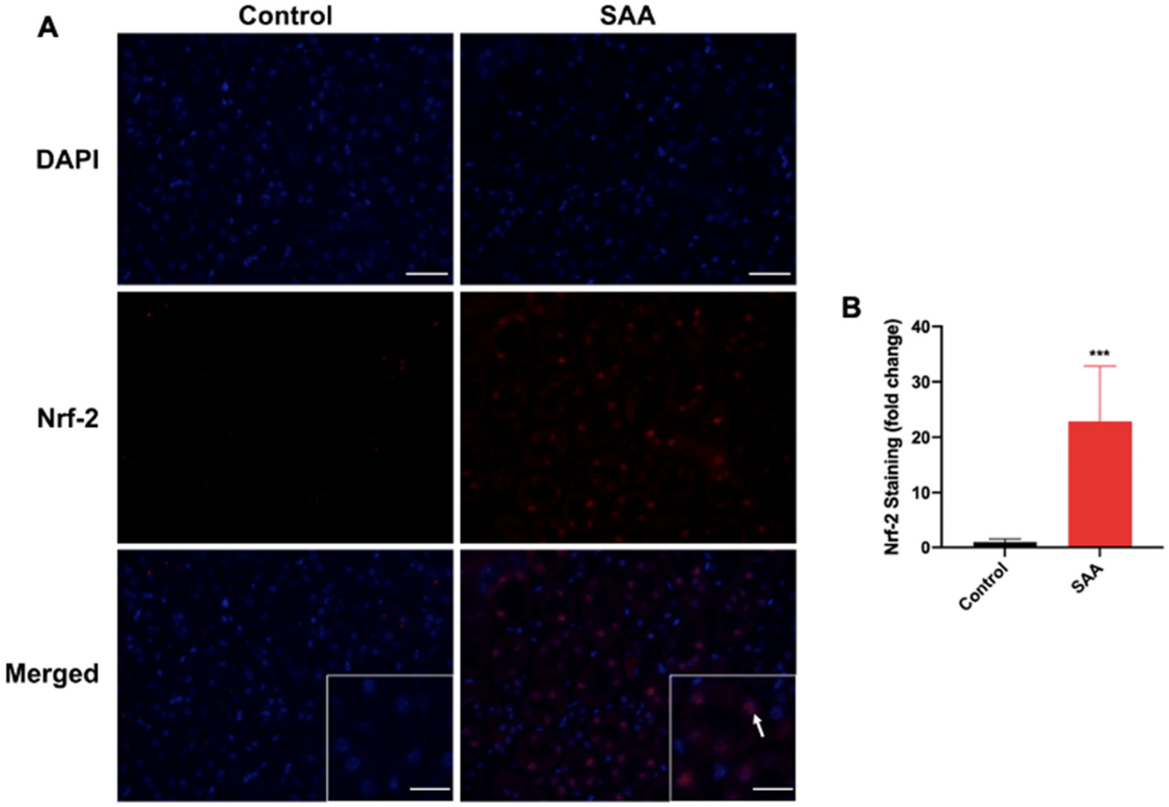

Figure S2

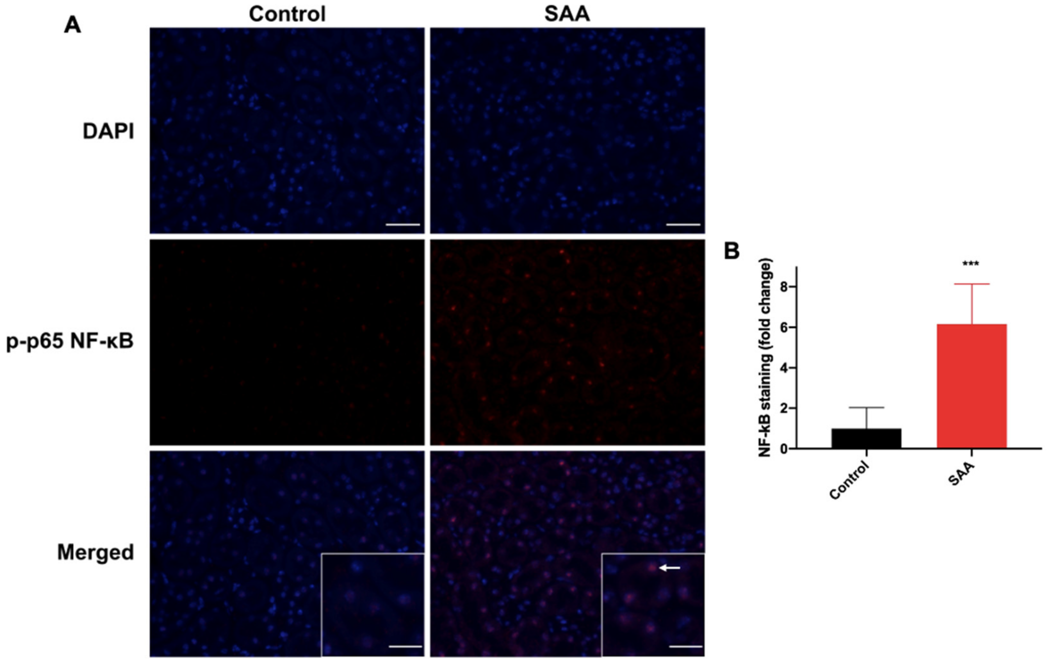

Figure S3

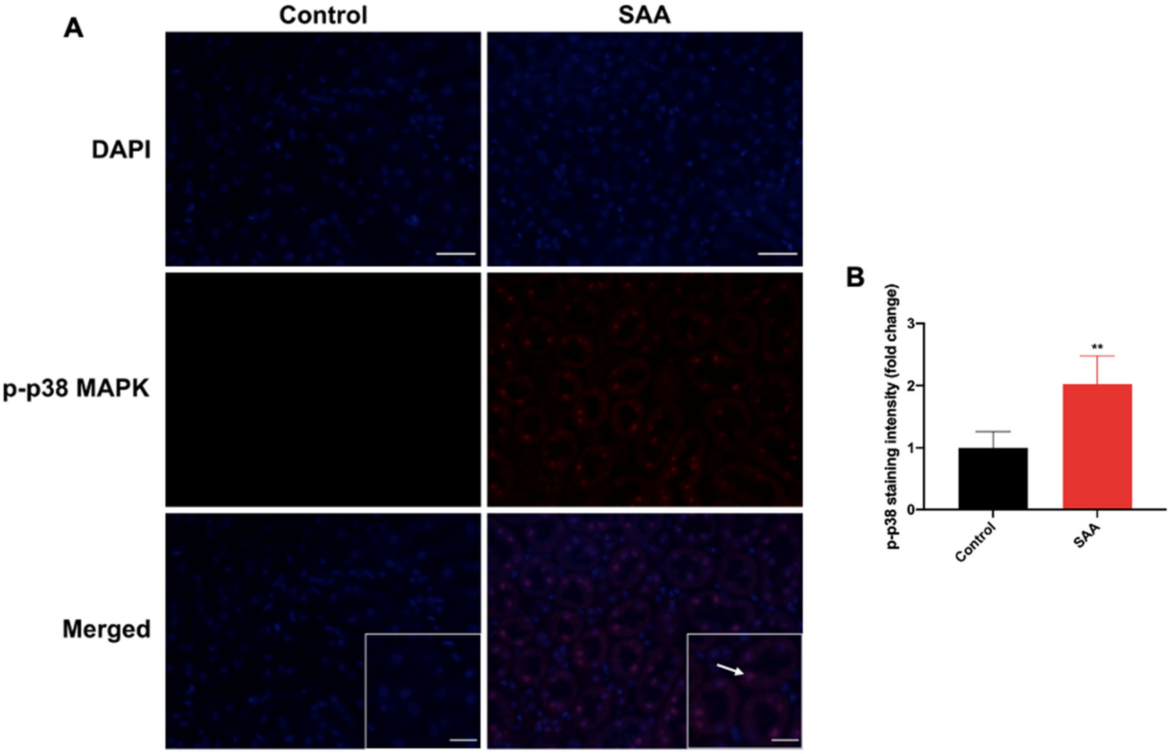

Figure S4

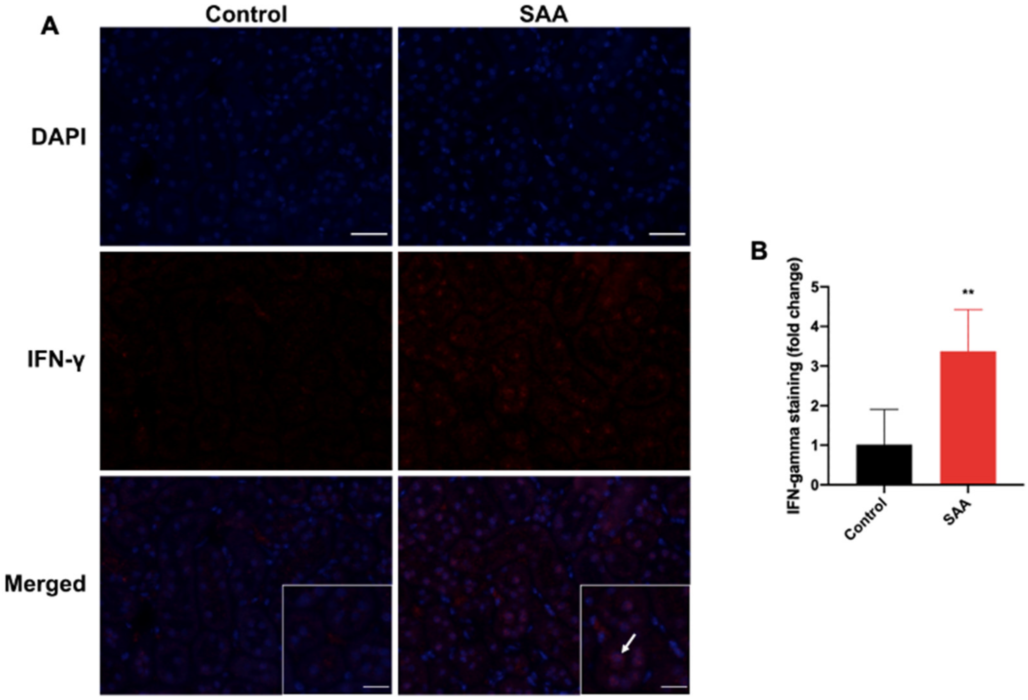

Supplement: Supplementary file 1 [file ijms-22-12582-s001.zip › ijms-1462439-supplementary.pdf]
